# Up-regulation of RNF187 induces hepatocellular carcinoma cell epithelial to mesenchymal transitions

**DOI:** 10.18632/oncotarget.22056

**Published:** 2017-10-19

**Authors:** Song-Lin Yu, Jin-Cai Wu, Peng-Fei Liu, Kai Liu, Chun Ye, Kai-Lun Zhou, Zhuo-Ri Li, Ya-Ping Xu

**Affiliations:** ^1^ Department of General Surgery, Tongji Hospital, Tongji University School of Medicine, Shanghai 200065, China; ^2^ Department of Hepatobiliary Surgery and Organ Transplantation, Hainan Provincial People's Hospital, University of South China, Haikou 570311, China; ^3^ Department of Gastroenterology Shanghai Tenth People's Hospital, Tongji University School of Medicine, Shanghai 200072, China

**Keywords:** hepatocellular carcinoma, RNF187, EMT, prognosis

## Abstract

Ring finger protein 187 (RNF187) has been identified to be a co-activator linking c Jun to Ras signaling. However, the expression and function of RNF187 in hepatocellular carcinomas (HCC) remains unclear. Here, we tried to determine the expression and roles of RNF187 in hepatocellular carcinomas (HCC).The expression of RNF187 was determined in HCC tissues and cell lines, and we found that RNF187 expressed highly in HCC tissues compared with the corresponding adjacent liver tissues both in mRNA and protein level, which was consistent with the result of immunohistochemistry on HCC tissue microarrays. In HCC cell lines, the level of RNF187 was positively associated with the HCC cells metastatic potential. By the RNF187 interference and cDNA transfection, we showed that the high level of RNF187 induced the HCC cells invasion and metastasis both *in vitro* and *in vivo*, as well as the high ability of colony formation.Mechanistically, we detected the high level of RNF187 promoted cell scatter by inducing epithelial-mesenchymal transition (EMT). Clinically, the high level of RNA187 was significantly correlated with a malignant phenotype, including larger tumor size, multiple tumors, and microvascular invasion. Importantly, high level of RNF187 correlated with HCC patients' shorter OS and lower disease free survival rates than those with low level of RNF187. Our results revealed that elevated expression of RNF187 induced hepatocellular carcinoma cell epithelial to mesenchymal transitions, and represented a novel marker for predicting the poor prognosis of HCC.

## INTRODUCTION

Hepatocellular carcinoma (HCC) is the fifth commonest malignancy and the third leading cause of cancer-related deaths in the world [1]. Clinically, HCC is characterized by its high invasive ability, the high incidence of recurrence, and the finite availability of effective therapy [2]. To date, surgery is still the preferred treatment of HCC, but unfortunately multifocal progression and early distant metastases hinder surgical curative treatment in most HCC patients [3, 4]. Thus, an understanding of the molecular mechanisms underlying HCC metastasis and recurrence is urgently required for effective chemotherapy treatment of HCC patients.

Substantial research, that focused on tumor development, has revealed that the epithelial-mesenchymal transition (EMT) is an essential reversible process during tumor progression [5]. Initially, EMT is found to be a fundamental procedure involved in embryonic development during which epithelial cells lose their differentiated properties and gain mesenchymal characteristics [6]. Then, a plethora of studies delineated the important role of EMT in pathogenesis of metastasis in epithelial tumors [7]. Indeed, the EMT cell is commonly apparent at the invading margin of the tumor mass and is likely to mediate cellular detachment and eventual metastasis [8]. Moreover, induction of EMT in cancer cell lines with low metastasis potential results in the acquisition of high metastatic properties *in vitro* [9, 10]. Of note is that several new studies have further demonstrated that cancer cells undergoing EMT appeared to gain the ability to resist apoptosis, chemotherapy and immunotherapy, respectively and also acquire stem cell features [11–13], which further emphasizes the role of EMT in mediating cancer metastasis, and the value of uncovering the fundamental molecular mechanisms underlying EMT.

The ubiquitin-proteasome system regulates a wide range of physiological processes including signal transduction, proliferation and apoptosis [14]. The dysregulation of ubiquitination was found to be directly involved in human cancers including HCC and may function as oncogene or tumor suppressor [15]. For example, the overexpression of ubiquitin ligase E3C promoted HCC progression by regulating tumor cell EMT [16], and a level of ubiquitin-specific protease 7 accelerated p14^ARF^ degradation by deubiquitinating thyroid hormone receptor-interacting protein 12 and promoting HCC progression [17]. Ring finger protein 187 (RNF187, also known as RACO1 or RACO-1) is a RING domain-containing ubiquitin E3 ligase. Normally, RNF187 is unstable in unstimulated conditions due to K48-linked autoubiquitination, and is stable in nondegradative K63-linked ubiquitination by the competition of degradative K48-linked ubiquitination regulated by activation of the Ras pathway [18]. Recently, several studies have examined the functions of RNF187. For example, RNF187 depletion was found to reduce cellular proliferation and downregulate several growth-associated AP-1 target genes, such as cyclin-dependent kinase 1 (CDC2), heparin binding EGF like growth factor (HBEGF) and cyclinD1 [19]. Additionally, transgenic overexpression of RNF187 was shown to enhance intestinal tumor formation by inducing aberrant Wnt signaling and through cooperation with oncogenic Ras in colon epithelial hyperproliferation [20]. Although, the reports on RNF187 functions are very limited at present, especially in tumors, the existing data show that it may play an important role in tumorigenesis and development.

Here, we tried to determine the expression of RNF187 in HCC tissues and cell lines. The role of RNF187 in HCC cells was investigated both *in vitro* and *in vivo* by using RNF187 interference and cDNA transfection. Finally, the clinical significance of RNF187 expression was further analyzed using tissue microarray (TMA) in 209 patients with HCC.

## RESULTS

### The expression of RNF187 is elevated in human HCC and positively associated with HCC malignant phenotypes

**Figure 1:**
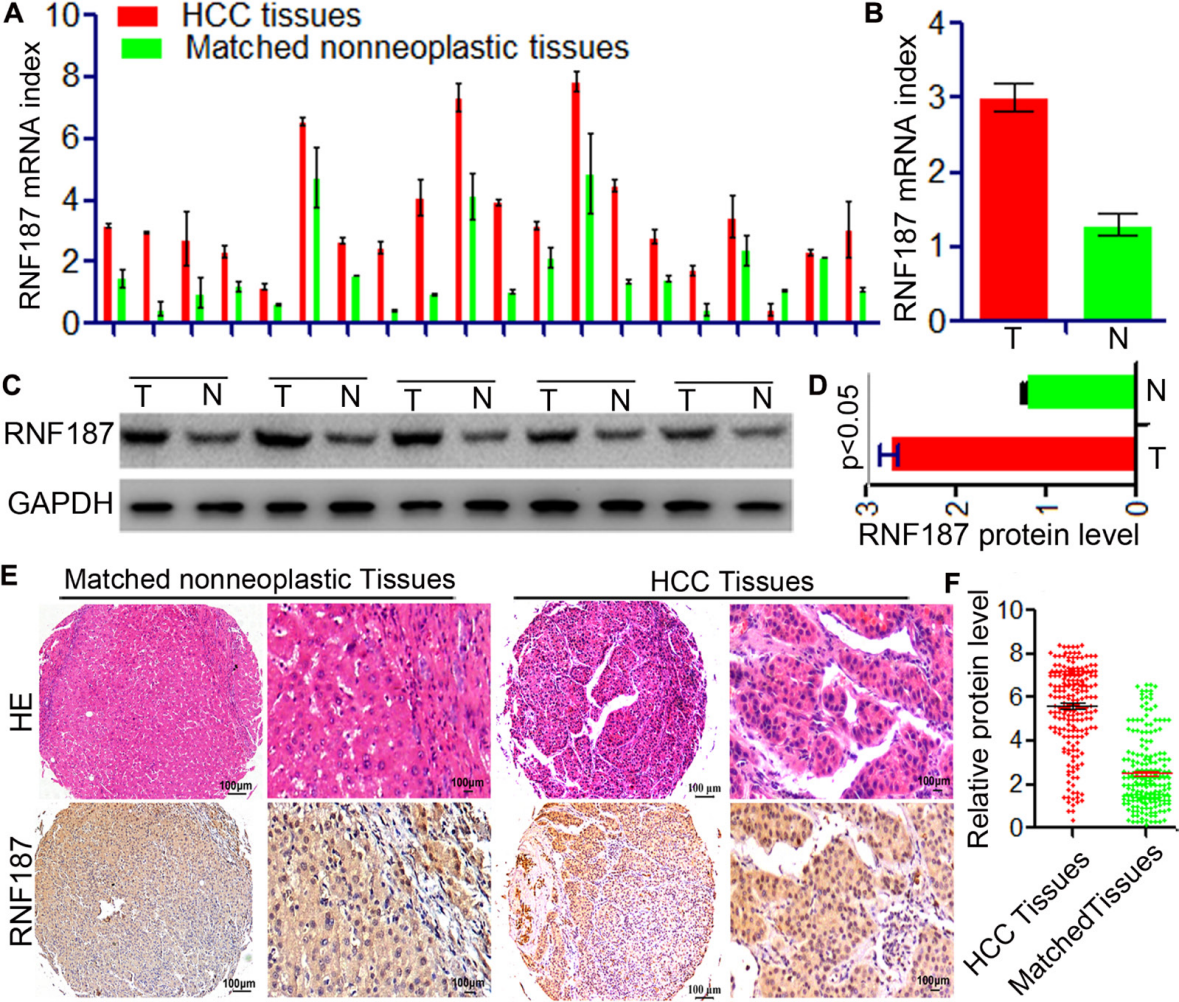
Up-regulation of RNF187 in HCC tissues (**A** and **B**) *q*RT-PCR showed that RNF187 mRNA level in HCC tissues was higher than that in adjacent non-tumorous liver tissues with statistically significant difference (*P* < 0.01); (**C** and **D**) Western blotting showed RNF187 protein expression in HCC adjacent non-tumorous tissues (*P* < 0.01); (**E** and **F**) Immunohistochemical staining demonstrated that expression level of RNF187 protein in HCC tissues was higher than that in adjacent non-tumorous tissues (*P* < 0.01).

Initially, the expression of RNF187 was determined by *q*RT-PCR and western blotting in 15 HCC and their matched peritumor tissues. The RNF187 expression was found to be higher in HCC than in matched peritumor tissues both at the level of mRNA (3.45 ± 0.29 *vs.*1.74 ± 0.13, *P* < 0.01, Figure 1A and 1B) and protein (2.75 ± 0.09 *vs.* 1.24 ± 0.02, *P* < 0.01, Figure 1C and 1D). Next, we examined the expression of RNF187 by TMAs including 209 patients with HCC (Figure 1E and 1F). Immunohistochemical results revealed that RNF187 was located in the cell cytoplasm and nuclei of neoplastic cells and highly expressed in 94 cases with variable intensities (44.98%), while low level of RNF187 were found in 55.02% (low expression,115/209) tumor tissues.

In HCC tissues, RNF187^high^ was significantly correlated with microvascular/bile duct invasion (*P* = 0.003), high TNM stage (*P* = 7.64E-11), multiple tumor (*P* = 0.026), and large tumor size (*P* = 1.90E-13). However, other clinical characteristics including age, sex, HBsAg background, tumor differentiation, liver cirrhosis, preoperative serum alpha-fetoprotein (AFP), and Child-Pugh scores were not significantly related to the expression of RNF187 (Table 1). The above results indicate that high levels of RNF187 may promote HCC progression.

**Table 1:** Association of RNF187 expression with clinicopathological parameters of HCC patients

ClinicopathologicalParameters	Total	RNF187 Expression		*p* value
		Negative	Positive	
	209	115	94	
Sex				
Male	179	94	85	0.055
Female	30	21	9	
Age range,yr				0.403
≤ 50	93	48	45	
> 50	116	67	49	
Tumor numbler				0.026
1	166	98	68	
≥ 2	43	17	26	
Tumor size, cm				1.90E-13
≤ 5	122	93	29	
> 5	87	22	65	
TNM stage				7.64E-11
I–II	144	101	43	
III–IV	65	14	51	
Grade of differentiation				0.065
I-II	152	89	63	
III-IV	57	26	31	
Microvascular/ bile duct invasion				0.003
Positive	59	23	36	
Negative	150	92	58	
HBX				0.491
Positive	167	94	73	
Negative	42	21	21	
Cirrhosis				0.855
Positive	187	104	83	
Negative	22	11	11	
AFP				1.000
≤ 400	131	72	59	
> 400	78	43	35	
ALT,U/L				0.684
≤ 75	181	101	8580	
> 75	28	14	14	

Abbreviations and Note: HCC, hepatocellular carcinoma; AFP, alpha-fetoprotein;TNM, tumor node metastasis; HBsAg, hepatitis B surface antigen; ALT, alanine aminotransferase; NS, not significant; The X^2^ test was used for comparison between groups. 50% for RNF187.

### Elevated expression of RNF187 is associated with increased invasion potential of HCC cells

**Figure 2:**
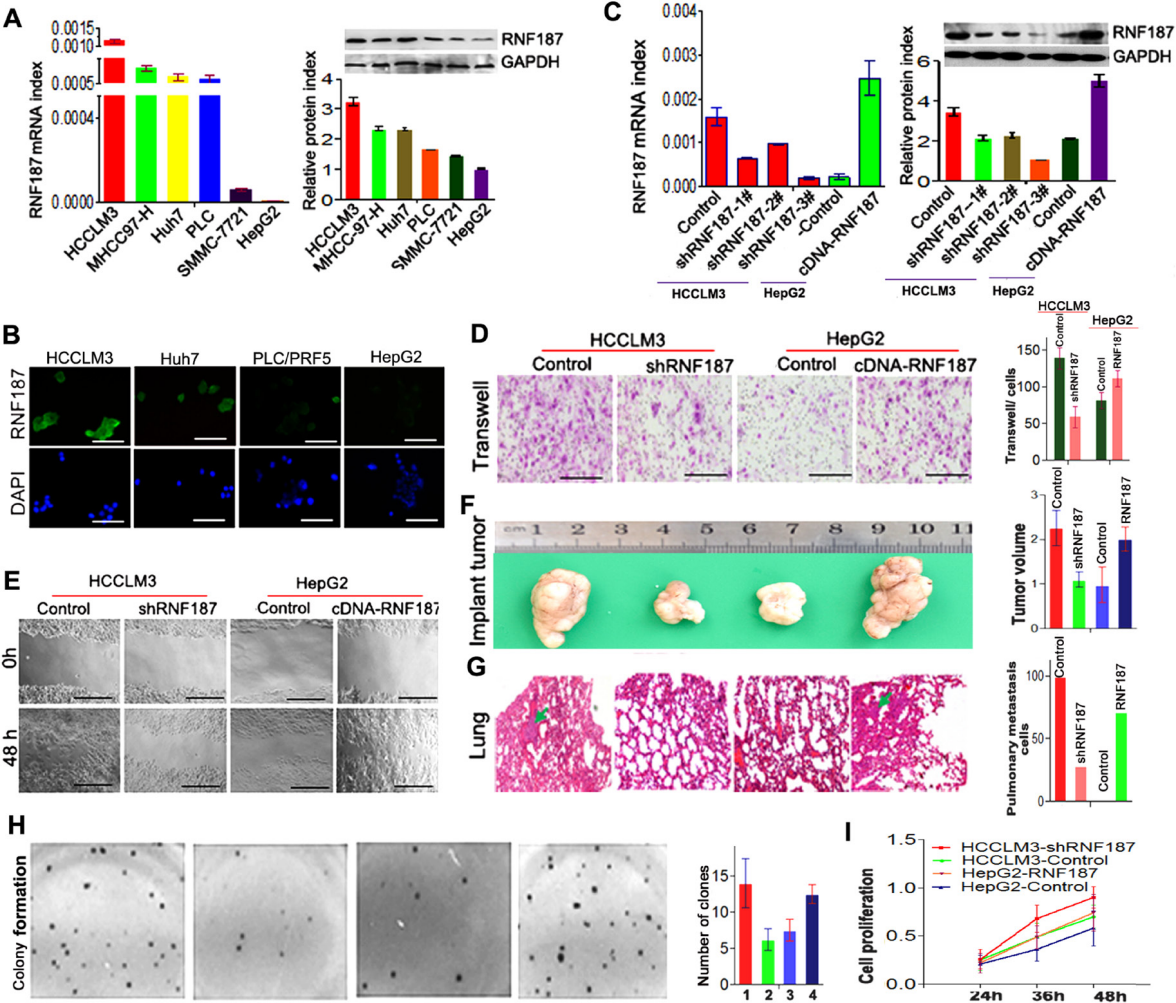
High level of RNA187 promote HCC cells invasion and metastasis both *in vitro* and *in vivo* (**A**) *q*RT-PCR showed RNF187 mRNA in HCCLM3, MHCC97-H, Huh7, PLC, SMMC-7721 and HepG2 cell lines; (**B**) Western blotting showed RNF187 mRNA in HCCLM3, MHCC97-H, Huh7, PLC, SMMC-7721 and HepG2 cell lines; (**C**) Immunofluorescent staining for RNF187 in HCCLM3, Huh7, PLC and HepG2 cells. DAPI stain (blue) was used to identify nuclei (Scale bar: 200 μm); (**D**) RNF187 expression was down-regulated by pGPU6-GFP-vshRNA-RNF187s in HCCLM3, and up-regulated by pGPU6-RFP-cDNA-RNF187 in HepG2 cells, and #3 was validated for the most efficient interference of RNF187 by qRT-PCR and immunoblotting. (**E**) Cells with low RNF187 expression migrated slowly compared with those with high RNF187 expression at 48 h for wound channel closure. (**F**) The clone formation was elevated in cells with high level of RNF187. (**G** and **H**) Transwell assay showed that the numbers of invading cells in groups with low RNF187 expression were higher than those with high RNF187 expression, and the volume of the tumors derived from HCC isogenic cell lines was calculated *in vivo* for 6 weeks; Serial sections from mouse lung showed the metastasis ability of cancer cells expressing different RNF187 (Scale bar: 50 μm); and cell proliferation was positively associated with RNF187 expression *in vitro*. Magnification, ×200 (E and G) and ×100 (I).

Here, we further sought to address the role of RNF187 in HCC cells. As shown in Figure 2A, the HCCLM3, MHCC97-H, Huh7 and PLC/PRF/5 cells expressed high levels of RNF187, while SMMC-7721 and HepG2 cells expressed low levels of RNF187 both in mRNA and protein levels. The immunofluorescent staining also showed that HCCLM3 and Huh7 cells expressed high level of RNF187, and we detected only low levels of RNF187 in PLC/PRF/5 and HepG2 cells (Figure 2B).

Next, we silenced RNF187 in HCCLM3 using specific vshRNAs and elevated RNF187 expression in HepG2 cells by cDNA-RNF187 transfection to investigate the effect of RNF187 on cell invasion and apoptosis. Among the 3 candidate shRNAs tested, shRNA-2 (designated shRNF187) was shown to be most efficient, and was therefore selected for subsequent studies (Figure 2C). By transwell assay, we found that the interference of RNF187 in HCCLM3 cells inhibited the invasion of HCCLM3 cells, while the up-regulation of RNF187 in HepG2 cells enhanced the invasion of HepG2 cells (Figure 2D). Using the wound healing assay, we showed that the RNF187 expression in HCC cells was positively associated with the migration of HCC cells (Figure 2D), which concurred with the *in vivo* examination (Figure 2F and 2G). Additionally, we also showed the ability of clone formation was elevated in cells with high level of RNF187 (Figure 2H), and the MTT assay showed that the knockdown of RNF187 expression in HCCLM3 cells significantly inhibited cell activity, while the forced RNF187 expression upregulated the HepG2 cell activity (Figure 2I).These results indicated the high levels of RNF187 was associated with an increased metastatic potential of HCC cells.

### High expression of RNF187 induces HCC cell EMT

**Figure 3:**
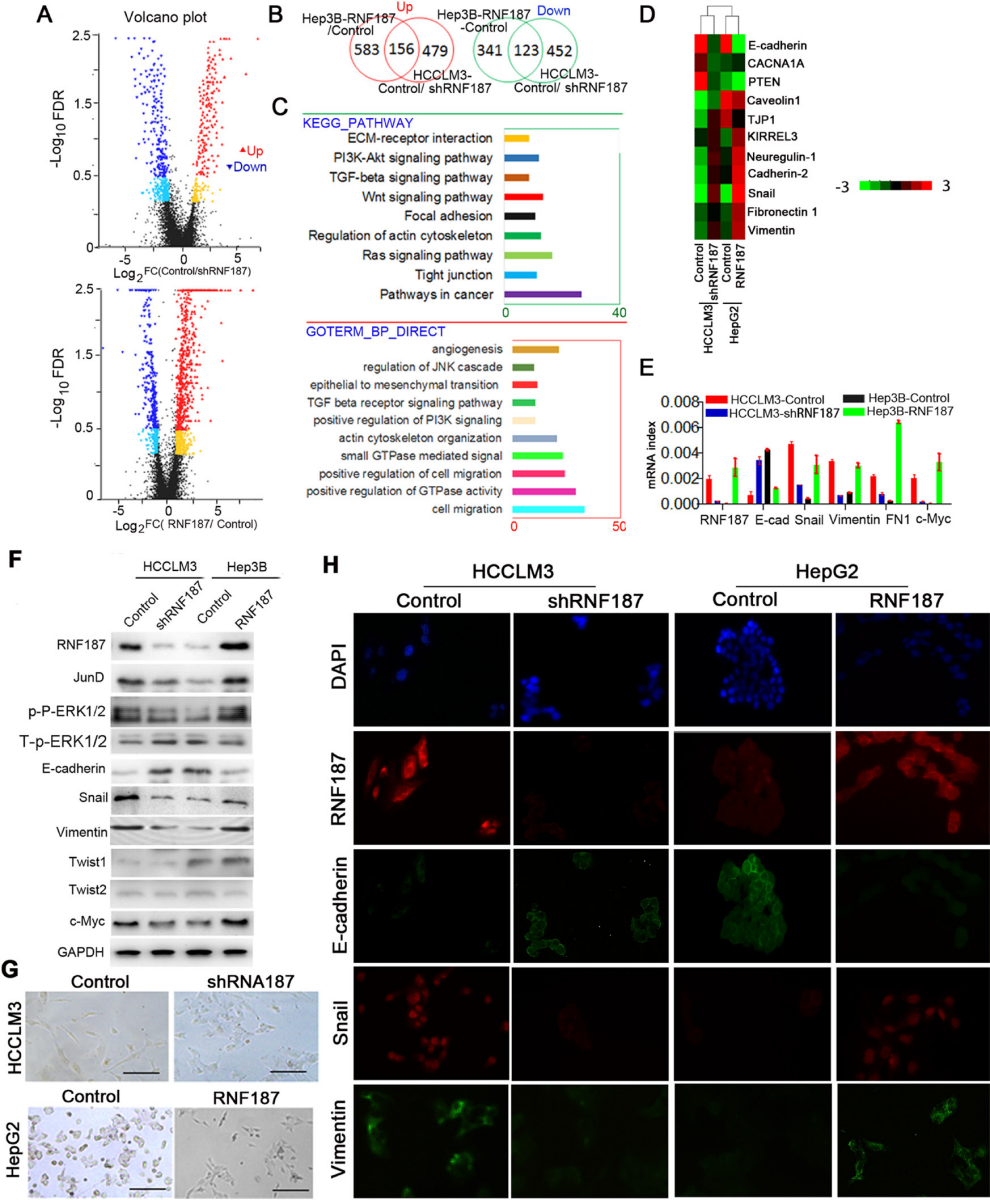
RNF187 overexpression induces an EMT in HCC cells (**A**) The RNA-sequence scatter plot showed the up or down-regulated gene between Hep3B-Control/ Hep3B-RNF187 cells and HCCLM3-Control/HCCLM3-shRNF187 cells detected by RNA-Sequence; (**B**) The overlap genes with up or down-regulation between Hep3B-Control/Hep3B-RNF187 cells and HCCLM3-Control/HCCLM3-shRNF187 cells; (**C**) The high level of RNF187 in HCC cells majorly involved in the regulation of signaling pathway related to cancer, such as ‘‘Pathway in cancer’’, ‘MAPK signaling pathway’, ‘epithelial-mesenchymal transition’, ‘Wnt signaling pathway’, and “Tight junction”; (**D**) The hotmap of RNA-seq showed the up-regulation and down-regulation of gene related to EMT; (**E**) Different expression of the epithelial and mesenchymal markers, as well as the transcription factors and c-Myc were showed between high and low expression RNF187 cancer cells (HepG2-control *vs*. HepG2-RNF187 and HCCLM3-control *vs*. HCCLM3-vshRNF187 cells; (**F**) Western blotting determined RNF187, E-cadherin, FN1, Vimentin, Snail, Twist1, Twist2, and c-Myc proteins expressiom in HCC cells with different TNF187 expression; (**G**) Phase-contrast microscopy showed the shapes of HepG2-Mock, HepG2-RNF187, HCCLM3-Mock and HCCLM3-vshRNF187. DAPI stain (blue) was used to identify nuclei (Scale bar: 200 μm); (**H**) Immunofluorescent staining for RNF187, E-cadherin, Vimentin and Snail in HepG2-mock, HepG2-RNF187, HCCLM3-mock and HCCLM3-shRNF187. DAPI stain (blue) was used to identify nuclei (Scale bar: 200 μm).

To delineate further the molecular basis by which RNF187 overexpression induces the invasion of HCC cells, whole genome transcriptome analysis on HCCLM3-control, HCCLM3-shRNF187, HepG2-control and HepG2-RNF187 cells were performed using RNA-seq (Figure 3A). By defining a threshold of the average FPKM ≥ 1, and the cut-off as more than a 2 fold change, 123 down-regulated genes and 156 up-regulated genes were found to be changed in both groups (Figure 3B), which mainly enriched cell migration, EMT, positive regulation of GTPase activity, actin cytoskeleton organization, positive regulation of PI3K signaling, Wnt signal pathway, TGF beta receptor signaling pathway by Gene Ontology (GO) analysis, and Kyoto encyclopedia of genes and genomes (KEGG) analysis also indicated they activate pathways involved in cancer, Ras signaling pathway, tight junction, regulation of actin cytoskeleton, etc (Figure 3C). Here, we further compared the epithelial, mesenchymal marker (Figure 3D), c-Myc and adherent molecules. As shown in Figure 3E, the E-cadherin mRNA was downregulated in cells overexpressing RNF187, while Snail, Vimentin , FN1 and c-Myc were upregulated, which was further validated by western blotting (Figure 3F). Then, we examined the cellular morphology of HCCLM3-control, HCCLM3-shRNF187, HepG2-control and HepG2-RNF187 cells, and found a distinct morphological difference was observed between cells expressing high level of RNF187 and low level of RNF187 (HCCLM3-control *vs.*HCCLM3-shRNF187, HepG2-control *vs.* HepG2-RNF187), and cells expressing a low level of RNF187 presented the typical cobblestone-like appearance of normal epithelium, while cells expressing a high level of RNF187 took on a spindle-like, fibroblastic morphology (Figure 3G). Then, we probed these cells with epithelial and mesenchymal markers by immunofluorescent staining. As shown in Figure 3H, HCCLM3-control and HepG2-RNF187 cells exhibited the typical EMT phenotype, including downregulation of E-cadherin and upregulation of vimentin. The above results collectively indicated a high level of RNF187 contributes to HCC cells invasion by inducing cell EMT.

### Clinical significance of RNF187 in the prognosis of HCC patients

**Figure 4:**
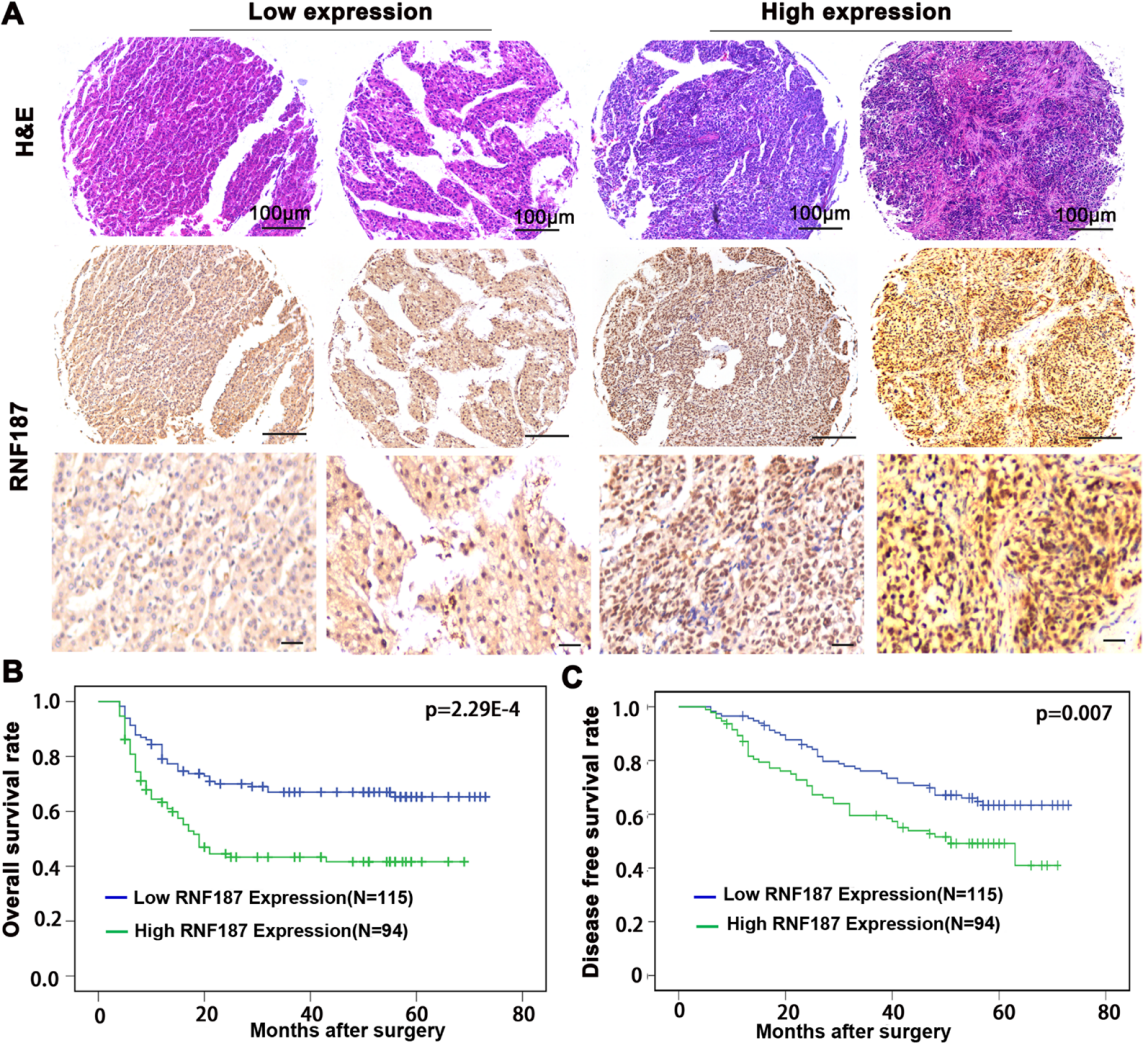
RNF187 overexpression is associated with the poorer survival of HCC patients (**A**) The representing of expression of RNF187 in HCC tissues (Scale bar = 200 μm); (**B** and **C**) RNF187 overexpression correlates the poorer survival and lower DFS rate of HCC patients.

To explore the association of RNF187 expression in HCC tissues with disease prognosis, patients were dichotomized into RNF187^high^ (moderate and strong; *n* = 94) or RNF187^low^ (negative and weak; n = 115) groups (Figure 4A). Statistically, there was a striking inverse association between RNF187 intensity and overall survival (OS; *P* = 2.29E-4) and to a less extent disease-free survival (DFS; *P* = 0.007) (Figure 4B and 4C).

Univariate analysis revealed that tumor diameter, tumor differentiation, tumor numbers, microvascular/bile duct invasion, TNM stage and RNF187 expression were predictors for OS and DFS (Tables 2 and 3), In a multivariate Cox proportional hazards model, TNM stage and tumor differentiation were independent prognostic factor for OS; vascular invasion,TNM stage and tumor differentiation were independent prognostic factor for DFS. Therefore, RNF187 expression is a valuable predictor for OS in patients with HCC.

**Table 2:** Univariate and multivariate analysis of prognostic factors of OS

Variable	Univariate		Multivariate	
	HR (95% Cl)	*p* value	HR (95 % Cl)	*p* value
RNF187 expresssion (positive vs negative)	1.766(1.157–2.696)	0.008	0.810 (0.478–1.375)	0.436
Tumor diameter (cm) (≤ 5 vs > 5)	1.725 (1.133–2.628)	0.011	0.805(0.395–1.409)	0.367
Tumor number (1 vs. ≥ 2)	3.018 (1.917–4.749)	1.81E-6	1.099(0.570–2.121 )	0.778
Vascular invasion (positive vs negative)	2.392 (1.555–3.679)	7.21E-5	1.453(0.857–2.464)	0.166
AFP (ng/ml) (≤ 400 vs > 400)	1.427 (0.906–2.248)	0.125		n.a.
TNM stage (I-II vs III-IV)	6.461 (4.170–10.01)	6.63E-17	4.922(2.759–5.621)	5.63E-8.
Tumor differentiation	5.608 (3.625–8.674)	9.43E-15	3.478(2.085–5.495)	8.13E-7
Sex (male vs female)	1.776 (0.891–3.544)	0.103		n.a.
Liver cirrhosis (positive vs negative)	0.748 (0.345–1.620)	0.462		n.a.
ALT (U/L) (≤ 50 vs > 50)	1.003 (0.545–1.846)	0.993		n.a.
Age (years) (≤ 50 vs > 50)	1.766 (0.891–3.544)	0.103		n.a.
HBsAg (positive vs negative)	0.817 (0.491–1.358)	0.435		n.a.

Abbreviations and Note: HCC, hepatocellular carcinoma; AFP, alpha-fetoprotein; TNM, tumor node metastasis; HBsAg, hepatitis B surface antigen; ALT, alanine aminotransferase; n.a., not applicable; The X^2^ test was used for comparison between groups. 50 % for RNF187.

**Table 3:** Univariate and multivariate analysis of prognostic factors of DFS

Variable	Univariate		Multivariate	
	HR (95%Cl)	*p* value	HR (95% Cl)	*p* value
RNF187 expresssion (positive vs negative)	2.137 (1.404–3.253)	3.97E-4	1.142 (0.687–1.900)	0.608
Tumor diameter (cm, ≤ 5 vs > 5)	1.110 (1.055–1.167)	5.24E-5	1.252 (0.696–2.176)	0.476
Tumor number (1 vs. ≥2)	1.618 (1.100–2.380)	0.015	1.251 (0.673–2.328 )	0.478
Vascular invasion (positive vs negative)	2.544 (1.674–3.867)	1.22E-5	1.594 (0.883–2.416)	0.025
AFP (ng/ml, ≤ 400 vs > 400)	1.151 (0.810–1.263)	0.160		na
TNM stage (I–II vs III–IV)	4.211 (2.756–6.433)	2.94E-11	2.332 (1.304–4.171)	0.004
Tumor differentiation	3.273 (2.147–4.990)	3.53E-8	1.809 (1.096–2.985)	0.002
Sex (male *vs.* female)	1.441 (0.766–2.708)	0.257		n.a.
Liver cirrhosis (positive vs negative)	0.677 (0.313–1.465)	0.322		n.a.
ALT (U/L, ≤ 50 vs > 50)	0.684 (0.343–1.362)	0.280		n.a.
Age (years, ≤ 50 vs > 50)	1.236 (0.809–1.889)	0.327		n.a.
HBsAg (positive vs negative)	1.057 (0.623–1.791)	0.838		n.a.

Abbreviations and Note: HCC, hepatocellular carcinoma; AFP, alpha-fetoprotein; TNM, tumor node metastasis; HBsAg, hepatitis B surface antigen; ALT, alanine aminotransferase; n.a., not applicable; The X^2^ test was used for comparison between groups. 50 % for RNF187.

## DISCUSSION

Here, we firstly report that the RNF187, a RING domain-containing ubiquitin E3 ligase, has an elevated expression in HCC tissues compared with the corresponding adjacent normal liver tissue both in mRNA and protein levels. In HCC tissues, RNF187 expression exhibits considerable heterogeneity in different samples. Moreover, the high levels of RNF187 were frequently found in tissues of malignant phenotypes, such as microvascular/bile duct invasion, high TNM stage, multiple tumors, and large tumor sizes, which indicates a high level of RNF187 functions as a promoter of HCC. Congruously, we demonstrated in both *in vitro* and *in vivo* models that overexpression of RNF187 promotes HCC by inducing HCC cells EMT. Importantly, a high level of RNF187 was found to correlate with poor survival of HCC patients. The above results indicate that high levels of RNF187 promote HCC invasion and metastasis.

Ubiquitination is one of the most abundant protein modifications and is shown to regulate a wide variety of biological processes, such as proteasomal degradation, endocytosis, subcellular localization, and kinase activation [21–23]. Ongoing work revealed that the disruption of ubiquitin pathways leads to the development of human diseases, including many types of tumors [24]. For example, many RING finger E3s, including the anaphase promoting complex (APC), MDM2 proto-oncogene (MDM2), breast cancer associated gene 1 (BRCA1), calcineurin B-like proteins (CBLs), and von Hippel-Lindau tumor suppressor (VHL), have been demonstrated to be implicated in either the suppression or the progression of cancer [25, 26]. Here, we have shown that RNF187 overexpression functions as a promoter in HCC invasion and metastasis, which is supported by the following evidence. Frist, a high level of RNF187 was positively associated with the metastatic potential of HCC cells. Second, a high level of RNF187 induces HCC cell EMT. Third, data from HCC patients demonstrated that overexpression of the RNF187 protein was significantly correlated with malignant phenotypes including larger tumor sizes, multiple tumors, and microvascular invasion, which are well established poor prognostic parameters.. Finally, clinical data demonstrated that HCC patients with RNF187 overexpression had poorer OS. In fact, using a modified yeast two-hybrid screen, RNF187 was originally found to be a novel JunD co-activator that links growth factor and oncogenic Ras signalling to the activation of AP-1 [27]. In view of the acknowledged proto-oncoprotein of JunD [28, 29], it is not unreasonable to draw the conclusion that a high level of RNF187 is a promoter of tumor progression.

At present, EMT is believed to be a vital process contributing to the tumor invasiveness and metastatic, including HCC [30, 31]. Mechanistically, the hyperactivation of PI3K/Akt and Raf/MAPK in EMT have been well demonstrated [8]. For example, Cryab overexpression fosters tumor progression in HCC by inducing cells EMT through activating the ERK signaling [32]. Here, we also detected an alteration in MAPK signal pathway associated with upregulation or downregulation of RNF187 by RNA-sequence. In HCC tissues, the positive immunostaining of ERK1/2 phosphorylation was increased by up to 45% [33], which is nearly in lines with the high level of RNF187 detected by immunohistochemistry in our study. The explanation might be due to the MEK/ERK pathway strongly promoting Lys 63-linked ubiquitylation of RNF187, which antagonized Lys 48-linked degradative auto-ubiquitylation of the same Lys residues [18]. In addition, we found that the forced expression of RNF187 in HCC cells could upregulate the level of c-Myc, which has been reported to be a powerful inducer of tumor cell EMT [6, 7]. In brief, high level of RNF187 promote HCC progression by inducing cell EMT, which closely related to the MAPK signal pathway.

In summary, our results collectively characterize overexpression of RNF187 as a major contributor to the invasion-prone phenotype of HCC, and define RNF187l as a chemotherapy target for HCCs.

## MATERIALS AND METHODS

### Cell lines and plasmids

HCC cell lines with different metastatic potentials (HCCLM3, MHCC97-H, Huh7, PLC/PRF/5, SMMC-7721 and HepG2) [3] were purchased from the Chinese Academy of Sciences. These cell lines were routinely maintained as the previous report [34].

The vshRNAs were constructed and synthesized by Shanghai Genomeditech Co., Ltd. The target sequence is 5ʹ-TGTGATGGACCGT AGGAAGAATTCAAGAGATTCTTCCTACGGTCCAT CAttttttc-3′. All transfections were performed as described previously [32].

### Patients and specimens

A total of 209 pathologically confirmed HCC patients, who received curative resection in Hainan Provincial People's Hospital, University of South China and Tongji Hospital, Tongji University between January 2002 and December 2010 were enrolled in the study. The inclusion criteria and patients standardized follow-up procedures have been previous documented [32]. Primary endpoints were disease free survival (DFS) and overall survival (OS) defined as the time intervals between the date of surgery and first report of tumor recurrence or patient death.

### RNA extraction and the quantitative real-time polymerase chain reaction (qRT-PCR)

HCC cells, HCC and matched peritumoral samples were employed to analyze the RNF187 mRNA levels using qRT-PCR. Total RNA was extracted using the TRI Reagent (Invitrogen, USA) according to the manufacturer's protocol. Complementary DNA (cDNA) was synthesized from 2 μg of total RNA using HieffTM First Strand cDNA Synthesis Super Mix (Yeasen, China). Amplification and detection were performed with 1 μl cDNA and HieffTM qPCR SYBR^®^ Green Master Mix (Yeasen, China). Glyceraldehyde 3-phosphate dehydrogenase (GAPDH) was used as the internal standard. Primers were RNF187: 5′-TGGAAATCATGAGAACTTG-3′ and: 5′-ACGGTCCATCACGTGTCC-3′; GAPDH: 5′-GGCAT CCTGGGCTACACTGA-3′ and 5′-GTGGTCGTTGAGGGCAATG-3′. The relative expression of RNF187 mRNA was analyzed by the comparative cycle threshold (Ct). All experiments were repeated in triplicate.

### Western blot analysis and immunofluorescence

Proteins extracted from HCC cells and samples were applied to detect RNF187 protein expression by western blotting as previously described [32]. The polyclonal rabbit anti-RNF187 (1:1000; Abcam, USA), polyclonal rabbit anti-JunD (1:500; Abcam, USA), Rat monoclonal to E-cadherin (1:5000; Abcam, USA), Rabbit polyclonal to snail (1:500; Abcam, USA), and Mouse monoclonal to Vimentin (1:1000; Abcam, USA) antibodies were used as the primary antibody, respectively. GAPDH (1:5,000; Chemicon, USA) was used as the internal control.

Polyclonal rabbit anti-human RNF187 (1:100, Abnova, China), rat monoclonal to E-cadherin (1:500), rabbit polyclonal to snail (1:100), and mouse monoclonal to Vimentin (1:100) antibodies were used for immunofluorescence. Nuclei were counterstained with 4’, 6-diamidino-2-phenylindole (DAPI, Vector Laboratories, Burlingame, CA).

### Matrigel invasion, colony formation assay, and *in vivo* metastasis assays

The matrigel invasion assay was carried out as follow. Briefly, 1 × 10^5^ cells tumor cells suspended in Dulbecco's modified Eagle's medium (DMEM) / RPMI 1640 supplementing 0.1% fetal bovine serum (FBS) were added to the up chamber. The supernatant of containing NIH3T3 was added into the down chamber. After incubation for 48 h, the invasive cells were counted under a Leica DMLA light microscope (Leica Microsystems, Wetzlar, Germany) as described [3]. The colony formation assay was performed as the previous report [28]. All experiments were carried out in triplicate.

3 × 10^6^ HCC cells were suspended in 100 ml DMEM and Matrigel (BD Biosciences, 1 : 1), and then inoculated into the liver parenchyma of nude mice after opening up the abdomen under anesthesia, and monitored once every 5 days for 45 days. The animals were killed under anesthesia and the visceral organs including the lungs and liver were examined by histopathological methods. Serial sections of mouse lungs were prepared and the number of lung metastases counted under a Leica DMLA light microscope (Leica Microsystems, Wetzlar, Germany) as the previous report [32].

### Construction of tissue microarrays (TMA) and immunohistochemistry

Tissue microarrays were constructed as described in an earlier study [3]. Immunohistochemistry (IHC) was performed using a two-step detection system (Dako, Carpinteria, CA) as previously described. Briefly, formalin-fixed and paraffin-embedded sections with a thickness of 4 μm were dewaxed in xylene and graded alcohols, hydrated, and washed in PBS. after microwave antigen retrieval (12 min in sodium citrate buffer, pH 6), the endogenous peroxidase was inhibited by 0.3% H_2_O_2_ for 30 min and the sections were incubated with 10% normal goat serum for 30 min. Primary antibodies (RNF187, 1:100) were applied overnight in a moist chamber at 4°C, followed by incubation for 30 min with the secondary antibody (RE7112, Novolink Polymer). The sections were developed in 3,3′-diaminobenzidine solution under microscopic observation and counterstained with hematoxylin. Negative control slides in which the primary antibodies omitted, were included in all IHC assays. The intensity of RNF187 was classified as high and low expressions (RNF187^high^ > 50% of tumor section, and RNF187^low^ ≤ 50%, respectively).

### RNA sequence and analysis

Total RNA was isolated from HCC cells and tissues using TRI Reagent (Invitrogen, USA) according to the supplier's protocol. The quality of RNA was evaluated using an Agilent 2100 Bioanalyzer (Agilent , Germany). cDNA synthesis was carried out according to the manufacturer's instructions, with the analysis performed by Majorbio (Shanghai, China). RNA-Seq reads were aligned to the human reference sequence National Center for Biotechnology Information (NCBI) hg19 with TopHat2 to calculate the values of fragments per kilobase of transcript per million mapped reads for known transcripts (FPKM). A two-class comparison of the pool of RNF187 high and the corresponding RNF187 low cell was performed to determine differentially expressed genes. We selected a fold change of 2 as the threshold for upregulation or downregulation, with a *P*-value < 0.05 of the fold-change being considered to be statistically significant.

### Statistical analysis

Statistical analyses were performed with SPSS 19.0 for Windows (Chicago, IL, USA) in this study. The RNF187 expression is described as means ± standard deviations. The student's *t*-test was employed for comparison between groups of normally distributed data. The overall survival (OS) and disease-free survival (DFS) rates were assessed by the Kaplan-Meier analysis. A Cox's proportional hazards regression model was used to analyze the independent prognostic factors. A *P*-value < 0.05 was considered to be statistically significant.

## Abbreviations

HCC: hepatocellular carcinoma
EMT: epithelial-mesenchymal transition
RNF187: ring finger protein 187
qRT-PCR: quantitative real-time polymerase chain reaction
cDNA: complementary DNA
shRNA: short hairpin RNA
FN1: fibronectin 1
MDM2: MDM2 proto-oncogene
BRCA1: breast cancer associated gene 1
CBLs: calcineurin B-like proteins
VHL: von Hippel-Lindau tumor suppressor
TMA: tissue microarray
DFS: disease-free survival
OS: overall survival
AFP: alpha-fetoprotein
APC: anaphase promoting complex
KEGG: Kyoto encyclopedia of genes and genomes
GO: gene ontology
FPKM: fragments per kilobase of transcript per million
CDC2: cyclin dependent kinase 1
HBEGF: heparin binding EGF like growth factor
